# Conceptual Model for the Integration of Marketing Strategies and Biomedical Innovation in Patient-Centered Care: Mixed Methods Study

**DOI:** 10.2196/77115

**Published:** 2026-01-06

**Authors:** Atantra Das Gupta, Yashpal Yadav

**Affiliations:** 1 Department of Biomedical Engineering, Nims University Rajasthan, NH-11C Delhi-Jaipur Highway, Jaipur, 303121, India, 91 7412077144; 2 Department of Electrical, Electronics & Electric Vehicle Engineering, Nims University Rajasthan, Jaipur, India

**Keywords:** biomedical technology, patient engagement, digital health, AI in health care, health marketing, wearables, CRM, customer relationship management

## Abstract

**Background:**

The increasing integration of biomedical technology and digital marketing is quickly transforming how patients engage with health care. The patient as an organization (PAO) model is explored in this study. The PAO model encourages patients to be active participants in health care decisions by leveraging wearables, mobile health (mHealth) apps, artificial intelligence (AI) platforms, and health care marketing strategies.

**Objective:**

This study aims to examine how the PAO model is evolving in practice and gain insight into both the opportunities and challenges created by the intersection of biomedical innovation and marketing practices in patient care.

**Methods:**

The scoping review was conducted across Scopus, Web of Science, PubMed, and Google Scholar. Selection criteria included articles published from 2014 to 2024. Studies were included if they examined connections among biomedical technologies, marketing strategies, and models of behavior and organizations. Studies lacking interdisciplinary focus or methodological rigor were excluded. Since this work was exploratory, it did not require a strict bias assessment. Additionally, findings derived from qualitative analysis of 18 semistructured interviews with patients, health care professionals, and technologists regarding their experiences with digital technologies and perceptions of trust, autonomy, and engagement were analyzed. Thematic analysis was applied to these interviews using open, axial, and selective coding.

**Results:**

From an initial pool of 22,740 records, 45 studies met the inclusion criteria and were analyzed. The review revealed that the integration of AI-based personalization, biosensors, and remote monitoring with marketing strategies, such as segmentation, customer relationship management systems, and behavioral nudging, offers potential to enhance patient autonomy and engagement. However, most studies were descriptive or exploratory, with limited empirical evaluation, particularly regarding ethical risks and digital inequality. Qualitative findings further illustrated how patients are adopting organizational behaviors, such as self-monitoring, real-time decision-making, and strategic management of health data. The following 5 key themes emerged: (1) patients as autonomous digital actors, (2) digital health as a behavioral ecosystem, (3) inequities in digital empowerment, (4) negotiating trust and ethical transparency, and (5) blended care as the preferred future. Although many participants embraced digital tools, concerns about data transparency, algorithmic bias, and loss of human connection highlighted important barriers to equitable adoption.

**Conclusions:**

The PAO model shows strong potential for personalizing care and engaging patients in health care. However, it is important to note that, so far, conceptual models have dominated the PAO literature, with little empirical evidence to support them. Therefore, as health care practices increasingly integrate digital technologies, it is crucial to develop appropriate safeguards for PAO models.

## Introduction

The concept of the patient as an organization (PAO) marks a significant shift in digital health care, redefining the patient as an active participant, strategic decision-maker, and key stakeholder in their care. Instead of being passive recipients of treatment, patients are increasingly managing their health data, engaging with providers, and shaping the design of the health care system. Based on organizational theory and health care strategy, this model encourages patients to take on roles typically held by structured entities, emphasizing self-management, participation, and governance. The World Health Organization (WHO) has recognized this change, calling for greater patient involvement in the development and implementation of health care systems to promote more responsive and effective care [1].

Despite its increasing importance, the PAO model remains mainly theoretical. Although benefits such as better health outcomes, lower system costs, and increased patient satisfaction are often cited, most practical efforts focus on mobile apps and wearable devices. However, truly decentralized health care, another major shift, depends on broader technological integration. Future systems will need to incorporate artificial intelligence (AI)–powered diagnostics, big data analysis, home-connected medical devices, and generative AI platforms such as ChatGPT [2]. These tools are not just extras; they change how health care is delivered, expanded, and customized.

Currently, patient-centered care tends to focus heavily on disease management. However, health care should encompass more than that: It should include wellness promotion, behavioral support, and preventive care [3]. This broader approach reflects a shift from reactive, “disease-care” models toward proactive, wellness-oriented digital health systems. The expanding role of mobile and digital platforms in areas like fitness tracking, lifestyle coaching, and preventive screening reflects this growth, creating not only new models of care but also new opportunities for health business innovation [4].

Within the PAO framework, the patient becomes a digitally connected and ethically engaged actor, actively managing their health records, co-designing service delivery, and even contributing to policymaking and research. This reframing introduces new dimensions of trust, transparency, and autonomy. It also brings the patient experience closer to the structure of advocacy-driven nonprofit organizations that represent patient and caregiver interests. However, trust is not simply a desirable outcome; it is essential, and yet, the ethical dimensions of digital health marketing, including privacy, consent, and algorithmic bias, are often underexplored [5].

The adoption of technology in health care has grown rapidly. More than 50% of patients now use telemedicine, more than 90% of care providers utilize electronic health records, and digital platforms such as social media are commonly used for health communication. However, these advances often mask ongoing digital inequalities along lines of race, geography, income, and education [6]. To address this, health care organizations should learn from business, particularly in behavioral segmentation, predictive analytics, and customer relationship management (CRM). Strategic planning and customized communication are crucial for expanding access, increasing reach, and improving health outcomes.

At the heart of this transformation lies biomedical technology: a fusion of biology, engineering, and computing designed to enhance care across the continuum. AI-powered imaging tools, biosensors, implantable monitors, and smart prosthetics now enable real-time diagnostics, adaptive treatment, and precision health management [7]. Examples include robotic-assisted surgeries that reduce risk, insulin pumps that automatically respond to glucose fluctuations, and wearable devices that track behavior and symptoms in real time, bridging gaps in traditional health care.

These technologies demand parallel evolution in data governance, security, and ethics. Management leaders must ensure that AI-powered systems comply with privacy regulations, cybersecurity frameworks, and inclusive design principles. The WHO’s *Global Strategy on Digital Health 2020‐2025* reinforces this direction, advocating for equity-focused digital transformation, especially in resource-constrained regions.

The PAO model is a convergence point, strategically integrating biomedical technology, behavioral science, and health care marketing. It supports a move from static, episodic treatment to dynamic, data-informed, and personalized health care management. Patients are no longer seen simply as users of care; they are empowered collaborators who coproduce health outcomes through technology-enabled engagement and decision-making [8].

Marketing frameworks provide a valuable lens for translating innovation into actionable steps. The traditional 4 Ps of product, price, place, and promotion take on renewed significance in the digital health era: [9]

- Product includes AI diagnostics, wearable biosensors, robotic interventions, and mobile point-of-care tools that support accuracy, personalization, and autonomy [10].
- Price is reflected in value-based care models like pay-for-performance, which reward quality and efficiency enabled through biomedical monitoring [11].
- Place reflects that care is no longer confined to clinical spaces. With portable, internet-connected tools, services can reach patients in their homes, remote regions, or emergency settings [6].
- Promotion through digital communication, including ethically designed AI messaging, social media campaigns, and CRM outreach, ensures that patients receive accurate, timely, and personalized health information [12].

The convergence of digital health technologies with health care delivery not only drives innovation but also supports global health equity. Scalable tools, like mobile diagnostics and cloud-based platforms, can extend care to underserved communities and help reduce disparities.

Although digital tools offer benefits such as improved access, personalized communication, and behavior change, deeper issues, like digital exclusion, ethical concerns, and systemic barriers, remain underexplored. Challenges such as unequal access, low digital literacy, and lack of trust persist, particularly in marginalized populations.

To address these gaps, this study combined a scoping literature review with qualitative research to examine the evolving concept of the PAO. We explored how patients increasingly engage in organizational-like behaviors, such as self-tracking, strategic participation, and co-creating care, while facing barriers related to equity, ethics, and infrastructure.

By grounding the PAO model in interdisciplinary and empirical research, we moved beyond theory to examine how emerging technologies, like AI tools, wearables, and mobile health (mHealth) apps, are reshaping patient roles. Our goal was to understand how these tools influence patient engagement, decision-making, and autonomy within connected, data-driven health care systems.

## Methods

### Mixed Methods Design

This study followed a mixed methods design consisting of 2 stages: Stage 1 involved a scoping review of the literature, and stage 2 included a qualitative study using semistructured interviews.

The study design was guided by the PRISMA-ScR (Preferred Reporting Items for Systematic Reviews and Meta-Analyses Extension for Scoping Reviews) framework and informed by constructivist-grounded theory principles to allow for the emergence of themes grounded in real-world patient experience.

### Stage 1: Scoping Review Method

#### Design and Framework

This study used a scoping review methodology guided by the PRISMA-ScR framework to ensure clarity, transparency, and academic rigor [13]. This review examined how biomedical technologies and marketing theory intersect within the emerging PAO model. Using a scoping review approach, we mapped contributions across health care, behavioral science, and management to highlight key insights and gaps. This helped build a clearer picture of how digital tools and health marketing can together support more responsive, data-driven, and patient-centered care.

#### Search Strategy and Data Sources

To achieve disciplinary depth and interdisciplinary breadth, an exhaustive academic search was conducted across 4 prominent databases: Scopus, Web of Science, PubMed, and Google Scholar. Boolean operators and carefully selected keyword combinations were used to explore the intersections between digital health, biomedical innovation, and health care marketing.

Key terms included *healthcare*, *CRM (Customer Relationship Management)*, *biomedical innovation*, *AI personalization in healthcare*, *digital nudging*, *behavioral economics in healthcare*, *health belief model, segmentation*, and *patient targeting*.

#### Research Questions

Research question (RQ) 1 was “In what ways do patients use biomedical technologies—such as wearables, mHealth apps, and AI-powered tools—to take control of their health and engage in self-management similar to organizational behavior, and how do ethical safeguards and equitable access shape these practices across diverse populations?”

RQ2 was “How are marketing strategies such as personalization, segmentation, and CRM integrated into biomedical technologies to enhance patient engagement, treatment adherence, and behavior change, and what equity and ethical challenges arise in this integration?”

RQ3 was “What social, structural, and contextual factors, such as digital literacy, access to infrastructure, and socioeconomic status, affect patients’ ability to use biomedical technologies effectively and equitably within the PAO framework?”

RQ4 was “How do patients understand and respond to ethical concerns associated with biomedical technologies, including data-driven personalization, algorithmic decision-making, and digital nudging, and how do these perceptions influence trust, autonomy, and willingness to adopt such tools?”

RQ5 was “How is the model of the PAO delivered by way of biomedical technologies, and how can it be conceived and regulated to give priority to equity, inclusiveness, and ethical integrity as core conditions for success?”

Together, these findings indicated that, although the literature offers rich descriptive themes, it often lacks rigorous appraisal of effectiveness and equity, which weakens the PAO model’s empirical foundation. This limitation informed the qualitative phase of this study, designed to provide deeper, evidence-based insights.

### Describing How the Scoping Review Informed the Qualitative Phase (From Stage 1 to Stage 2)

#### Overview

Although the scoping review offered a strong conceptual foundation, highlighting how digital tools and marketing strategies are shaping a new model of patient engagement, it also revealed important gaps. Many studies were exploratory and lacked insight into how patients in real life experience these innovations. To address this, we turned to the second phase of the study: qualitative interviews. This phase aimed to ground the theoretical potential of the PAO model in the voices and lived experiences of patients, clinicians, and digital health developers. By listening closely to how people interact with technologies in their everyday health routines, we were able to explore how the PAO model is beginning to take shape beyond theory and where its promises meet real-world complexity.

#### Research Design

**Figure 1.**
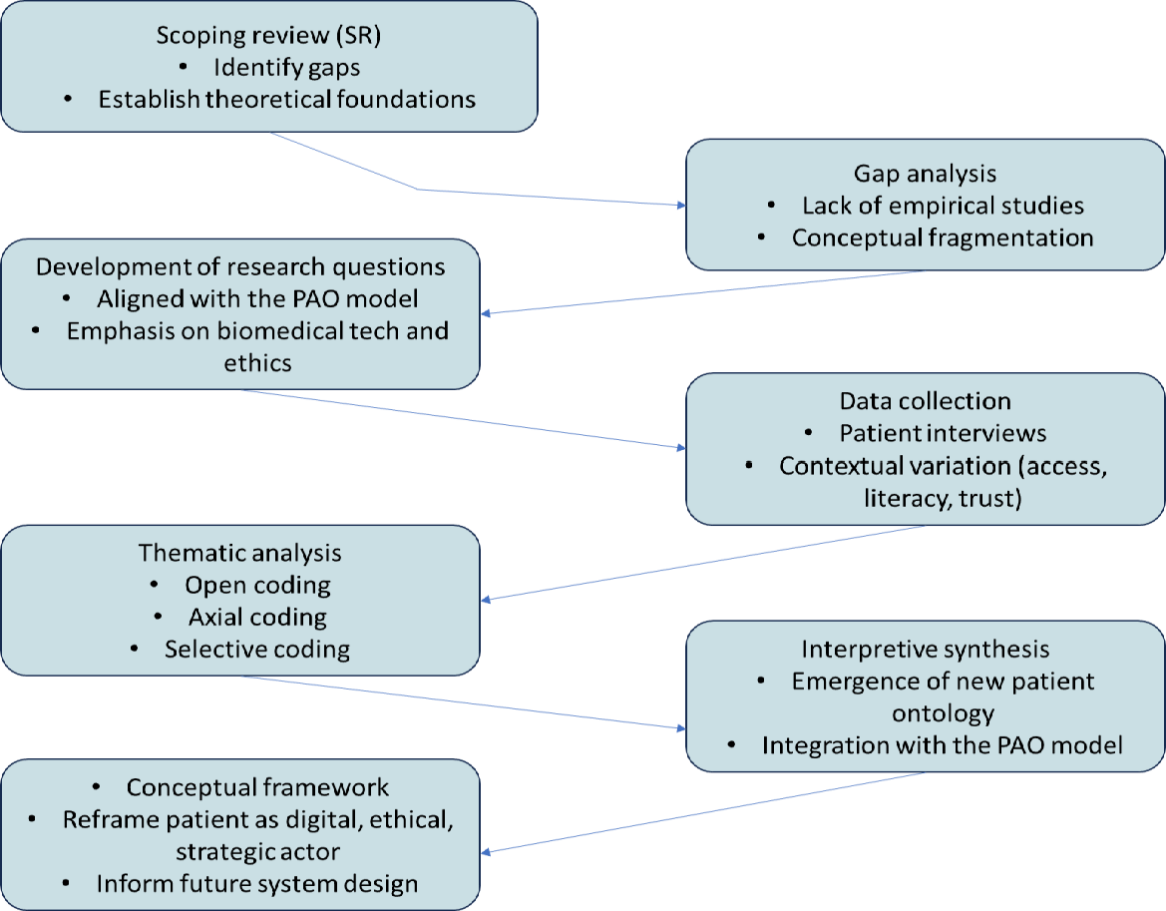
Methodological flowchart for developing and synthesizing the patient as an organization (PAO) conceptual framework based on the authors’ scoping synthesis and principles of qualitative research design.

Figure 1 illustrates a visual roadmap linking the scoping review to the qualitative phase of this study. The scoping review provided a foundation by highlighting key conceptual gaps, such as the lack of empirical evidence and fragmented definitions of the PAO. These insights directly guided the development of RQs centered on biomedical technology, ethics, and patient empowerment. Based on these questions, qualitative data were collected through in-depth patient interviews, focusing on real-world factors like digital access, literacy, and trust. Using a structured thematic analysis process, including open, axial, and selective coding, patterns that emerged were combined into a coherent framework. This integration of findings not only refined the PAO model but also helped develop a new patient ontology that portrays individuals as active, ethical, and strategic contributors to the future design of health systems.

### Stage 2: Qualitative Study Method

#### Overview

The qualitative study aimed to explore how patients, health care professionals, and biomedical technologists experience the integration of digital tools and marketing strategies in health care. Although the sample size (n=18) may seem small, it was intentionally chosen to align with the study’s exploratory goals and to prioritize conceptual depth and theoretical insight. Participants were purposively selected from 3 stakeholder groups to ensure diversity in experience, role, and digital exposure. Saturation was reached after 12 interviews, with additional participants confirming the existing themes rather than adding new ones. Beyond saturation, the adequacy of the sample is supported by the richness and variation in responses, which provided enough depth to identify recurring themes related to autonomy, trust, digital exclusion, and the changing role of the patient. The study prioritized analytical depth over statistical breadth, aligning with qualitative methods that focus on developing conceptual frameworks rather than producing generalizable results. Furthermore, because the study’s goal was to refine the emerging PAO model and examine its real-world applications, this approach enabled a layered, interpretive analysis of how digital tools influence patient behavior and engagement. Nonetheless, broader claims about the model’s applicability will require further empirical testing across larger, more diverse populations.

#### Qualitative Research Design

In today’s evolving health care ecosystem, the PAO concept lies at the center of transformation, shaped by advancements in biomedical technologies and strategic health care marketing approaches [14]. Marketing plays a crucial role in influencing how patients perceive, adopt, and integrate such technologies into their daily self-care routines [15]. However, adoption remains a nuanced process, shaped by factors such as technological trust, usability, privacy concerns, digital literacy, and the demand for accessible and personalized solutions.

This qualitative study was designed to explore the lived experiences of patients, health care providers, and biomedical technologists to understand the interplay between patient empowerment, biomedical innovation, and health care marketing strategies. Specifically, it sought to examine the facilitators and barriers to adoption of digital health technologies and identify how marketing can be aligned with the values and expectations of technologically enabled, self-managing patient communities.

#### Participants and Sampling

Participants were recruited using purposive sampling to ensure that they were relevant to the research focus. The inclusion criteria required participants to have a minimum of 1 year of experience using or developing digital health technologies, such as wearable biosensors, mHealth apps, or AI-powered tools. Participants represented 3 stakeholder groups: patients actively using digital tools, health care professionals implementing these technologies, and biomedical technologists involved in the design and deployment of these technologies. Individuals with no relevant experience or those unable to provide informed consent were excluded from the study.

#### Data Collection

We conducted 18 semistructured interviews using secure online video conferencing platforms to facilitate accessibility and geographic diversity. Each interview lasted between 45 minutes and 60 minutes and was audio-recorded with participant consent. The interview protocol included open-ended questions designed to elicit insights into technology adoption behaviors, trust dynamics, usability experiences, marketing communication preferences, and ethical concerns.

#### Data Analysis

The study used a grounded theory–inspired coding process, comprising open, axial, and selective coding, to identify patterns and themes within the data [16]. Interviews were transcribed verbatim and analyzed iteratively using qualitative analysis software. Thematic saturation was reached after 12 interviews, at which point adding new data no longer yielded novel insights. Consistent with the *Law of Diminishing Returns in Qualitative Research* [17], data collection ceased at this point to maintain methodological efficiency and thematic clarity.

This approach ensured that each interview contributed meaningfully to understanding how patient organizations engage with digital tools, what shapes their decision-making, and how biomedical solutions can be better aligned with trust, values, and behavioral drivers.

#### Ethical Considerations

The study was conducted in accordance with ethical guidelines for qualitative research. All participants provided informed consent. Data were anonymized, securely stored, and used solely for research purposes. Because the interviews were conducted for academic purposes only and will not be used for commercial or promotional purposes, review by an institutional review board was not required [18-20].

## Results

### Stage 1: Scoping Review Results

#### Mapping the PAO Landscape

The search process yielded a total of 22,740 records (Scopus: n=7462; Web of Science: n=5081; PubMed: n=6417; and Google Scholar: n=3780). Following the removal of 6505 duplicate entries, 16,235 unique records remained for initial screening. Abstract-level review excluded 9523 studies due to topic misalignment, lack of peer review, or language barriers. The remaining 6712 full-text articles were assessed for methodological quality and thematic alignment.

Of these, 142 studies met the inclusion criteria for the qualitative synthesis. However, 97 were excluded during deeper analysis because they lacked the conceptual integration required by the multiphase coding framework. Ultimately, 45 high-impact and thematically diverse publications were selected for inclusion.

**Figure 2.**
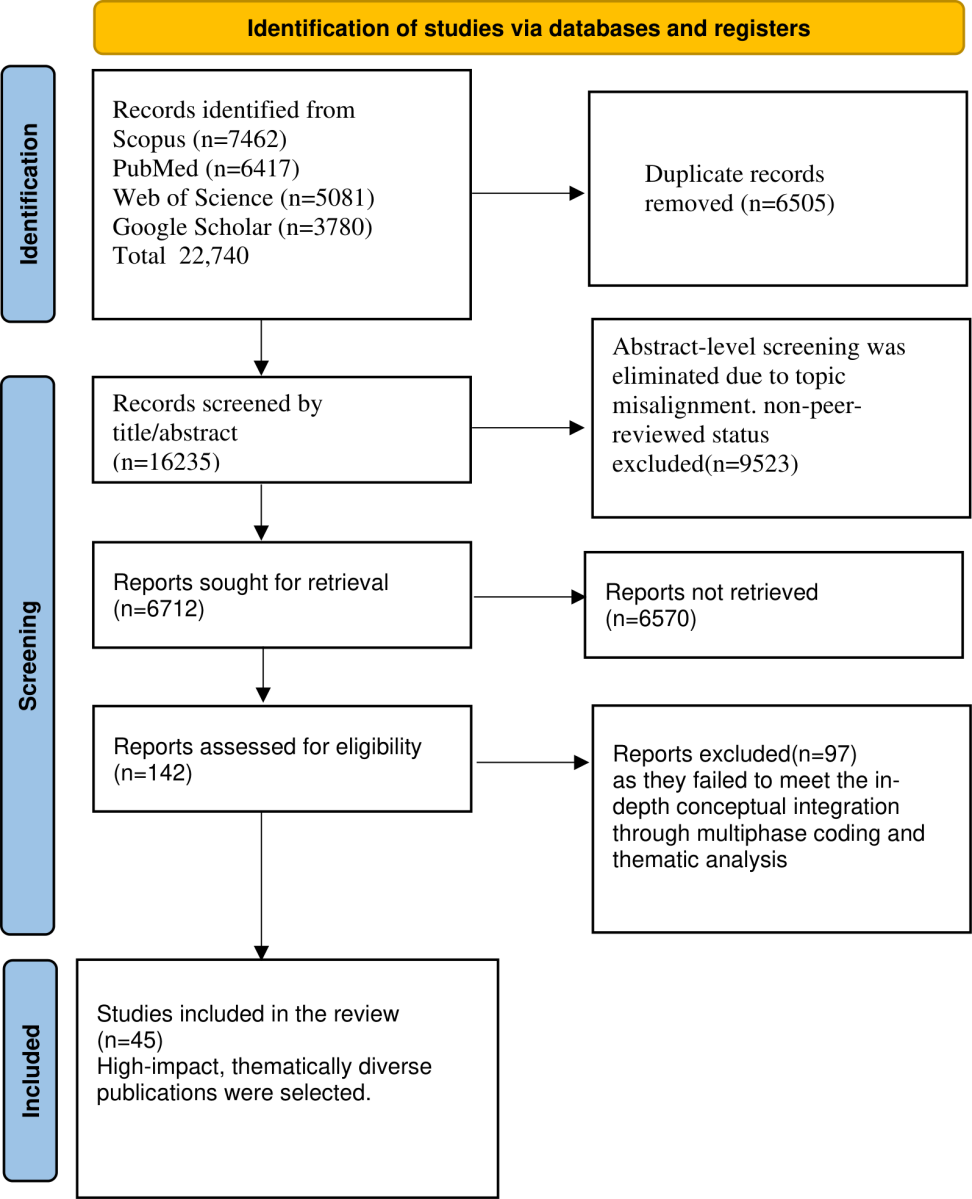
PRISMA-ScR (Preferred Reporting Items for Scoping Reviews and Meta-Analyses Extension for Scoping Reviews) flow diagram [21].

This entire process adhered to the PRISMA-ScR guidelines and is visually summarized in Figure 2, which outlines the identification, screening, eligibility, and inclusion phases of the review.

#### Scoping Review Findings: Mapping Biomedical and Marketing Integration Within the PAO Model

From over 22,000 records, the scoping review identified 45 key studies exploring how biomedical innovation intersects with health care marketing under the PAO model. Common themes included AI-driven segmentation, behavioral economics, CRM, and digital nudging, highlighting new ways to personalize care and boost patient engagement. Many studies also pointed to the growing use of wearables, biosensors, and mHealth platforms to support real-time feedback and predictive analytics.

However, the evidence was uneven. Most research was exploratory, based on small pilot studies or case studies, with few rigorous evaluations or long-term outcomes. Promising tools such as AI segmentation and CRM often failed to account for cultural, ethical, or trust-related factors. Digital nudging raised concerns around autonomy, but few studies tested its real-world impact.

In short, although the PAO model shows strong potential, its practical value is still emerging. Clearer evidence and stronger ethical frameworks are needed to turn these innovations into scalable, patient-centered solutions.

#### Three Core Themes Emerged Across the Literature

##### AI-Driven Personalization and Segmentation

Many studies examined how AI and predictive analytics are used to tailor interventions to individual health profiles. These approaches mirror commercial segmentation strategies, enabling more responsive, targeted care.

##### CRM and Engagement

The adaptation of CRM systems in health care is enabling more continuous and personalized communication between patients and providers. This has shown promise in improving satisfaction, adherence, and long-term engagement.

##### Digital Nudging and Behavioral Influence

Several articles discussed the use of behavioral nudges embedded in apps and digital platforms to encourage healthier choices. However, concerns around autonomy and the ethical boundaries of persuasive design were rarely explored in depth.

### Gaps in the Evidence Base

In addition to these thematic strengths, the review also highlighted important gaps in the current evidence base. Most studies were exploratory, drawing on pilot projects, descriptive analyses, or commercial analogies rather than longitudinal evaluations or controlled trials. Empirical testing of effectiveness, ethical risks, and digital equity was limited, particularly regarding diverse patient representation and co-designed solutions. Issues like data transparency, algorithmic bias, and unequal access to technology received minimal attention.

Despite these limitations, the review provided a rich conceptual foundation for understanding how the PAO model is developing. It also helped guide the qualitative phase of the study by identifying key mechanisms such as personalization, behavioral design, and engagement technologies through which patients are increasingly acting as strategic, data-informed participants in their care.

### Stage 2: Qualitative Findings—Patients as Self-Organizing Actors

#### Participant Overview

Interviews took place with 18 participants who had direct and indirect experience with patients and represented clinicians and digital health technology experts. The participants had various experiences with technologies such as AI-based health applications and patient portals.

#### Exploring Patient Experiences With Biomedical Technologies Through the PAO Lens

Table 1 shows how patient experiences and stakeholder insights informed the conceptual development of the PAO model across behavioral, ethical, structural, and systemic dimensions by mapping RQ1-RQ5 to the thematic categories that emerged during the qualitative analysis.

**Table 1.** Alignment of interview themes with corresponding research questions (RQs) in the patient as an organization (PAO) framework, developed by the authors based on thematic analysis of qualitative interview data.

Interview question	Related theme
RQ1	Patients as autonomous digital actors
RQ2	Digital health as a behavioral ecosystem
RQ3	Inequities in digital empowerment
RQ4	Trust and ethical transparency
RQ5	Blended care and systems-level PAO framework

#### How Do Patients Describe Their Experiences With Biomedical Technologies (eg, Wearables, Health Apps, AI Tools) for Monitoring, Managing, and Making Decisions About Their Health?

This explored self-regulation, autonomy, and organizational behaviors from the patient’s perspective. The interview focus was on “How do you use digital tools to track or manage your health? What role do these tools play in your decision-making?”

#### In What Ways Do Patients Perceive That Digital Health Platforms Apply Personalization, Nudging, or Targeted Communication Strategies to Influence Their Health Behaviors?

This investigated how marketing strategies are experienced through biomedical technology (eg, STP, CRM, and behavioral nudging). The interview focus was on “Do your apps or tools provide personalized suggestions or reminders? How do these affect your motivation or trust?”

#### What Challenges Do Patients Face With Accessing, Understanding, and Effectively Using Biomedical Technologies, Particularly Across Socioeconomic or Geographic Contexts?

This addressed the digital divide, equity, and structural limitations to PAO operationalization. The interview focus was on “What makes it easy or difficult for you to use digital health tools? How do factors like internet access or digital literacy impact your experience?”

#### How Do Patients View Consent, Data Privacy, and Transparency Issues When Interacting With AI-Powered or Data-Driven Biomedical Tools?

This explored ethical concerns tied to trust, data handling, and algorithmic personalization. The interview focus was on “How do your apps or devices use your health data? How does this affect your trust in the system?”

#### How Do Patients Envision the Ideal Balance Between Digital Technology and Human Interaction in Health Care, and What Features Do They Believe a Future Digital Health System Should Include?

This aimed to co-create or inform a future interdisciplinary PAO framework. The interview focus was on “How do you feel about relying on technology versus speaking with a health care provider? What would your ideal digital health system look like?”

#### Understanding the Operationalization of the PAO Model

##### Thematic Analysis

Thematic analysis of the qualitative interviews uncovered a set of interconnected themes that demonstrate how patients are increasingly adopting organizational-like roles within digital health ecosystems [22]. These themes corresponded to the core pillars of the PAO framework, specifically behavioral agency, digital engagement, structural equity, ethical trust, and systems-level integration.

As shown in Table 2, each theme was closely aligned with one of the core RQs (RQ1–RQ5), demonstrating how participants’ lived experiences reflect the evolving roles of patients as autonomous decision-makers, digital collaborators, and ethical stakeholders. From personalized technology use and behavioral self-regulation to trust in AI-driven tools and systemic challenges in access, the findings provide a nuanced understanding of how the PAO model is being enacted in real-world contexts.

**Table 2.** Thematic evaluation of biomedical technology within the patient as an organization (PAO) framework based on a thematic synthesis of selected literature in the scoping literature review by the authors.

Theme	Key studies (authors, year)	Role of biomedical technology	Critical evaluation	Thematic transition
Segmentation, targeting, and personalization (STP)	Brommels, 2020 [23]	AI^a^-driven segmentation tools and digital health communication platforms support tailored interventions.	Although conceptually strong, most evidence comes from small-scale or single-site pilots. Limited comparative testing undermines PAO’s claim to broad applicability and limits scalability.	It provides the foundation for targeted interventions but requires stronger, cross-system validation to serve as a reliable PAO mechanism.
Health belief model (HBM)	Ahadzadeh et al, 2015 [24]	AI-powered symptom checkers and telemedicine platforms tailor communication to patient risk perceptions.	Evidence is effective for literate and digitally fluent users but neglects populations with low health literacy or limited digital access. This exclusion undermines PAO’s inclusivity.	It establishes a psychological basis for personalization, but without testing in vulnerable groups, it risks reinforcing inequities within PAO.
Behavioral influence and social marketing	Evans, 2006 [25]	Behavioral analytics and health apps are designed for broad population-level engagement.	This demonstrates strong public health influence, yet applications in chronic care and low-resource contexts remain underexplored. The lack of contextual adaptation limits PAO’s scalability.	It informs engagement strategies but remains descriptive; long-term effectiveness must be empirically tested for PAO adoption.
Customer relationship management (CRM)	Mohiuddin, 2019 [26]	Predictive communication tools, EHR^b^-linked messaging, and reminder systems sustain engagement.	Although these are effective for engagement, the evidence draws heavily on commercial analogies. Few longitudinal health care studies exist, leaving CRM’s role in PAO sustainability unproven.	It suggests potential for trust-building and continuity but risks oversimplifying health care relationships unless tested in diverse care environments.
Branding and trust-building	Mohamed, 2022 [27]	Transparent design interfaces, ethical AI systems, and privacy protocols support trust.	Although this is conceptually robust, most studies are cross-sectional, offering little insight into how trust evolves. This gap weakens PAO’s ethical foundation.	It establishes an ethical entry point for PAO adoption, but without longitudinal studies, it remains more aspirational than practical.
Innovation adoption (diffusion theory)	Dearing and Cox, 2018 [28]	Wearables, telehealth tools, and peer-based adoption stories encourage uptake.	It explains early adoption effectively but overlooks structural barriers for marginalized groups. Evidence is skewed toward digitally privileged populations.	This drives momentum for mainstreaming PAO but requires inclusive adoption models to ensure equity.
Behavioral nudging and economics	Auf et al, 2021 [29]	Gamification, default settings, and subtle interface nudges are embedded in health apps.	These encourage short-term behavior change, yet few real-world studies examine ethical limits in high-stakes care. Weak empirical grounding risks compromising autonomy in PAO.	This supports digital habit formation but needs stronger ethical evaluation to avoid coercion in PAO practices.
Information-motivation-behavioral skills (IMB) model	Rongkavilit et al, 2010 [30]	Decision aids, chatbots, and adaptive mobile learning systems build skills and motivation.	It shows strong empowerment potential but most evidence comes from youth or disease-specific contexts (eg, HIV). Broader transferability has not been tested, limiting PAO’s reach.	It bridges education and empowerment but requires validation in chronic and multimorbidity settings for PAO credibility.
Patient empowerment and co-creation	Vainauskienė and Vaitkienė, 2021 [31]	Real-time feedback dashboards and participatory design platforms encourage collaborative care.	This demonstrates high potential for co-design, but most applications are exploratory or conceptual. Lack of real-world implementation reduces PAO’s structural legitimacy.	It completes the feedback loop for PAO but risks tokenism without evidence of genuine patient integration into decision-making.

a AI: artificial intelligence.

b EHR: electronic health record.

##### Patient-as-Agent: Emergence of Self-Regulation and Decision-Making

Participants consistently described themselves as “managers” of their health, citing wearables, symptom trackers, and health apps as tools that extend their decision-making processes. They reported scheduling appointments, adjusting lifestyle behaviors, and even questioning clinical advice based on insights derived from digital devices. This reflects a shift toward self-regulation, where patients assume roles once reserved for organizational actors, such as analysts, strategists, and communicators.

My smartwatch alerts me when my heart rate spikes, and I’ve learned to adjust my pace or diet accordingly. It feels like having a personal assistant, but ultimately, I take the final decision.

[Participant 11, age 65 years, rural man, retired government officer]

##### Digital Health as a Marketing System: Engagement, Nudging, and Feedback Loops

The integration of marketing concepts, particularly segmentation, nudging, and personalization, was evident in how participants responded to app interfaces and notifications. Many acknowledged that gamified elements, personalized reminders, and visual dashboards were crucial for sustaining motivation. However, responses also revealed ethical ambivalence: Although participants appreciated targeted support, they expressed concerns about potential manipulation and the use of data.

*The app rewards me for reaching my step goals, but I often question how it uses my data. Is it genuinely assisting or trying to sell me something?*

[Participant 10, age 30 years, urban woman, renowned corporate figure]

##### Digital Exclusion and Structural Constraints

Despite enthusiasm for digital tools, disparities were evident. Participants from lower socioeconomic backgrounds or remote locations reported limited access, connectivity challenges, and difficulties navigating complex interfaces. Older participants often lacked digital literacy or felt overwhelmed by “data overload.” This highlights the digital divide and the need for inclusive design.

*The hospital I visited in Delhi told me to use the app, but I don’t have Wi-Fi at home. And when I try to use it, it’s too confusing. I stop.*

[Participant 18, age 55 years, rural woman, school teacher]

##### Trust and Transparency in AI and Personalization

Participants articulated trust as both a prerequisite and an outcome of digital health interaction. When transparently delivered, personalized care enhanced patients’ perception of safety and value. However, algorithmic opacity and inconsistent recommendations undermined confidence. Many desired “explainable AI” and clearer data use policies.

*If I knew the logic of how it decides what to show me or suggest, I would trust it more. However, it feels like a black box right now.*

[Participant 2, age 40 years, urban man, real estate businessman]

##### Need for Human Touch Amid Digital Expansion

Although digital interfaces were valued for convenience and personalization, patients emphasized the irreplaceable value of human connection. Participants advocated for blended care models where technology augments clinician relationships but does not replace them.

I appreciate the app, but I prefer a human to explain serious issues rather than a chatbot.

[Participant 6, age 53 years, suburban woman, homemaker]

### Thematic Coding Framework: Operationalizing the PAO Model in Digital Health

To explore how the PAO model is unfolding in real life, we used a 3-step coding process rooted in constructivist-grounded theory. This approach helped us make sense of recurring patterns in what participants shared during interviews.

In the first stage (open coding), we identified specific behaviors and concerns—things like self-tracking habits, reactions to AI-driven personalization, responses to digital nudges, and worries about data privacy and trust (see Table 3). Commercial analogies helped illustrate the PAO model by translating strategies from retail, tech, and service industries to the individual patient level. These comparisons offer fresh perspectives on personalization, engagement, and collaboration, but their nonclinical origins highlight the need for stronger validation within health care settings.

**Table 3.** Commercial analogies in the patient as an organization (PAO) model developed by the authors drawing on practices from Amazon, Apple, IKEA, Netflix, and loyalty or supply chain models to illustrate conceptual parallels in the PAO framework.

Commercial practice	Application in the PAO model	What it offers	What it misses
Retail segmentation (eg, Amazon product recommendations)	AI^a^-driven patient segmentation using health and behavioral data to personalize interventions	Tailors care in real time, making treatment more responsive	It risks oversimplifying complex patient needs, and potential for algorithmic bias exists.
Customer loyalty programs (eg, airline frequent flyer, hotel rewards)	Health care CRM^b^ platforms that predict adherence and personalize communication	Builds long-term engagement and strengthens patient-provider relationships	Patients are not “customers”—trust in care requires ethical accountability, not just loyalty.
Digital nudging (eg, Netflix auto-play, app notifications)	Health nudges in apps prompting exercise, diet, or medication adherence	Encourages healthy habits and sustained engagement	It may compromise autonomy if patients feel manipulated rather than supported.
Co-creation in services (eg, IKEA design input, open-source platforms)	Participatory health platforms where patients co-design care plans and give feedback	Empowers patients as partners and fosters collaboration	Access barriers and digital literacy gaps may exclude vulnerable populations.
Branding in consumer tech (eg, Apple’s design and trust strategy)	Branding of digital health platforms to foster confidence and ease of use	Reduces anxiety and encourages adoption	Trust in health care must rest on transparency, fairness, and safety, not just design.
Supply chain logistics (eg, just-in-time inventory systems)	Wearables and biosensors providing continuous data for anticipatory care	Prevents crises through early intervention; improves efficiency	It relies on constant connectivity and raises concerns about privacy and governance.

a AI: artificial intelligence.

b CRM: customer relationship management.

The individual insights shown in Table 3 were then grouped into broader categories during axial coding (Table 4), including themes like digital self-governance, behavioral engagement tools, and trust and ethical friction.

**Table 4.** Open coding of everyday experiences with biomedical technology in the patient as an organization (PAO) context, developed by the authors based on qualitative interview data (2025).

Code	Description
Self-tracking	Use of apps or devices to monitor health metrics
Decision autonomy	Making health decisions based on digital feedback
AI^a^ personalization	Adjustments based on algorithmic insights
CRM^b^-based reminders	App notifications encouraging health behaviors
Gamification	Points, badges, and visual cues in apps
Behavioral nudging	Subtle prompts guiding patient behavior
Data confusion	Difficulty interpreting or trusting data
Lack of digital access	Limited or no access to the internet or devices
Low digital literacy	Challenges using digital tools due to the skills gap
Patient skepticism	Doubts about data privacy or app motives
Desire for human contact	Preference for in-person over digital interaction
Trust in tech	Confidence in digital tools and recommendations
Transparency concerns	Lack of clarity around data usage and AI processes

a AI: artificial intelligence.

b CRM: customer relationship management.

Finally, in the selective coding stage, we pulled everything together into 5 core themes (Table 5) that reflect how people are experiencing and adapting to digital health tools: (1) patients as autonomous digital actors, (2) digital health as a behavioral ecosystem, (3) inequities in digital empowerment, (4) negotiating trust and ethical transparency, and (5) blended care as the preferred future. These themes directly address the research’s central question: How is the PAO model operationalized in practice through biomedical technology tools, and what are the ethical, behavioral, and structural implications of this shift?

**Table 5.** Axial coding, with code families reflecting the patient as an organization (PAO) model and developed from thematic synthesis of interview data by authors (2025).

Code family	Constituent codes
Digital self-governance	Self-tracking, decision autonomy, AI^a^ personalization
Behavioral engagement tools	CRM^b^-based reminders, gamification, behavioral nudging
Structural barriers	Lack of digital access, low digital literacy
Trust and ethical friction	Data confusion, patient skepticism, and transparency concerns
Human-digital synergy	Desire for human contact, trust in tech

a AI: artificial intelligence.

b CRM: customer relationship management.

These themes showed how patients are taking a more active role in their care as well as how their experiences are shaped by access, design, trust, and support.

**Figure 3.**
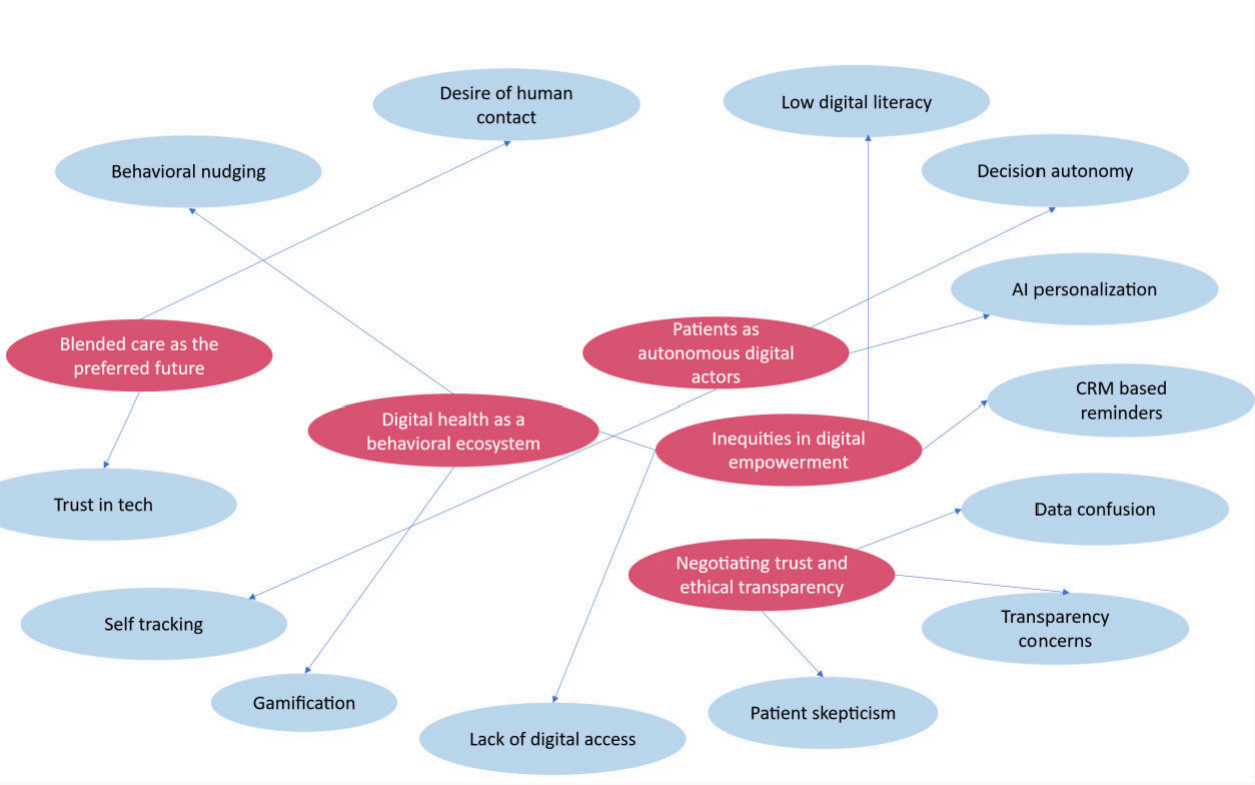
This code tree, conceptualized by the authors, outlines key patient-centered challenges and themes that shape the digital health experience and documents how individuals interact with emerging technology, the obstacles they face, and the ethical concerns that influence trust and adoption in diverse health care settings. AI: artificial intelligence; CRM: customer relationship management.

To bring this all to life, Figure 3 maps how individual experiences, like using gamified health apps or struggling with digital literacy, connect to the bigger picture. It visually traces how personal interactions with technology shape and are shaped by the evolving roles patients are playing in today’s health care systems.

This thematic framework grounds the PAO model in real-world voices and helps us better understand how digital tools and health strategies are redefining the patient experience (Table 6). The themes directly address the research’s central question: How is the PAO model operationalized in practice through biomedical technology tools, and what are the ethical, behavioral, and structural implications of this shift?

**Table 6.** Overarching themes and thematic insights linking patient experiences to the patient as an organization (PAO) framework, as developed from thematic synthesis of interview data by the authors (2025)*.*

Theme	Description	Links to PAO model
Patients as autonomous digital actors	Through digital interfaces, patients demonstrate growing self-regulation and strategic behavior, embodying organizational traits such as monitoring, evaluation, and adaptation.	Aligns with PAO’s redefinition of the patient as an active agent in their health journey
Digital health as a behavioral ecosystem	Apps, nudges, CRM^a^ reminders, and gamification work in tandem to shape sustained patient engagement. These resemble marketing systems and feedback loops.	Reflects the application of marketing theory (STP^b^, CRM) within health care
Inequities in digital empowerment	Access to PAO-enabling technologies is uneven, constrained by structural factors such as socioeconomic status, literacy, and infrastructure.	Reveals a key limitation in PAO implementation across diverse populations
Negotiating trust and ethical transparency	Patients demand clarity around data use and AI^c^ decisions. Depending on design and communication, digital systems can facilitate and undermine trust.	Essential for the PAO model to evolve into an ethically grounded framework
Blended care as the preferred future	Despite the convenience of digital technology, human interaction remains essential. Patients seek a hybrid model where technology augments, rather than replaces, the human touch.	Supports a flexible PAO model that integrates human empathy with technological precision

a CRM: customer relationship management.

b STP: segmentation, targeting, and positioning.

c AI: artificial intelligence.

The thematic structure provides a grounded, evidence-based map of how patients are beginning to embody organizational behaviors, where friction points exist, and what conditions are necessary for equitable and sustainable transformation.

### Visual Key: Linking Raw Data to Core Themes

In the thematic analysis shown in Figure 3, the red circles represent the high-level themes that emerged during selective coding. These themes, such as *patients as autonomous digital actors* and *blended care as the preferred future*, capture the broader conceptual insights that frame how the PAO model is realized in practice.

In contrast, the blue circles reflect the more granular codes identified during open coding. These codes, such as *self-tracking*, *gamification*, and the *lack of digital access*, are grounded in participants’ direct experiences and form the foundation of each theme.

Together, the red and blue circles illustrate how concrete participant narratives (blue) were synthesized into overarching patterns of meaning (red), offering a clear line of sight from real-world observations to theoretical insight. This visual structure is central to understanding the layered complexity of the PAO framework.

### Thematic Categories (Axial Coding)

Within the data gathered from the interviews, 5 key themes were evident (Table 5).

The first was the use of digital self-governance. Patients took charge of managing their conditions without assistance by using technology to monitor and track progress.

The second, behavioral engagement tools, covered how reminders, gamification, and nudging led to habit development but also posed questions about user autonomy.

Trust and ethical friction represented the third theme. Attitudes to trust and ethical friction ranged from uncertainty about AI decisions to data use concerns and digital manipulation.

Structural barriers, the fourth theme, included problems such as low digital literacy rates, lack of device and internet access, and poor application usability presented challenges to digital tool use.

In the fifth theme, human-digital synergy involved the use of many valuable digital resources but with an emphasis on augmentation rather than replacement in human interaction.

### Integration to Core Themes (Selective Coding)

These factors were integrated to form 5 thematic areas (Table 5) giving insight into the practical application of the PAO model: (1) patients as autonomous digital actors, (2) digital health as a behavioral ecosystem, (3) inequities in digital empowerment, (4) negotiating trust and ethical transparency, and (5) blended care as the preferred future.

These 5 themes formed a complex set of understandings about how people interact with digital health care, not only as technology users but also as strategic and emotionally committed actors. The relationships among these themes are shown in Figure 3, which illustrates how lower-level observations (blue nodes) relate to higher-level organizational themes (red nodes). This level of mapping highlights the richness and depth of analysis in this study and how individual experiences are part of a broader trend in organizational behavior.

## Discussion

### Principal Findings

#### Defining the Conceptual Boundaries of the Field

This scoping review advanced the PAO model from a metaphor into a strategic framework rooted in biomedical technology, marketing theory, and digital health practice. Rather than viewing patients as passive care recipients, the literature highlights their role as active, data-aware decision-makers who interact with and shape digital health ecosystems.

The PAO model operates across 3 levels. At the *micro level*, patients manage their health using tools such as wearables, apps, and AI-driven feedback to support self-monitoring and daily decision-making. At the *meso level*, marketing strategies, such as CRM, segmentation, and co-creation, structure how patients engage with health care providers and systems. At the *macro level*, broader issues like policy, regulation, and digital equity influence access, trust, and the overall impact of digital health transformation.

Although the model offers a rich, multilayered view of modern health care, it also presents conceptual challenges, particularly around how individual behaviors translate into system-wide change and how societal structures shape personal experiences. Instead of seeing these tensions as limitations, the review positions them as opportunities to refine the model.

By connecting data flows and decision-making across individual, organizational, and societal levels, the PAO framework has the potential to become a cohesive, ethical, and scalable approach to digital health, one that centers the patient while addressing the realities of technology, governance, and equity.

#### From Static Segmentation to Dynamic Personalization

Segmentation, targeting, and positioning (STP) have long been foundational marketing strategies. In health care, these have evolved using AI-powered segmentation based on real-time physiological and behavioral data. Studies by Brommels [23] and Minvielle et al [32] described how biomedical technologies enable the continuous reclassification of patients into highly personalized cohorts. However, ethical challenges, particularly those related to algorithmic bias and the risks of overpersonalization, remain underexplored.

#### Repositioning Behavioral Theories Through Technology

To truly connect biomedical technology with marketing in health care, we need more than comparisons; we need a clear, practical framework. Although strategies such as personalization, CRM, and nudging are often paired with tools like AI and wearables, their true impact on patient behavior is rarely examined.

A better path is to combine behavioral models with real-time tech—using tools that respond to patients’ habits, motivations, and health concerns. However, this isn’t just a design question; it’s an ethical one too. When does a helpful nudge become manipulation?

For the PAO model to be meaningful, it must do more than describe trends. It should explain how these elements work together in everyday care and ensure that technology supports, rather than controls, the patient experience.

#### Rethinking CRM for a Nonlinear Health Journey

CRM models, which have traditionally been used to foster patient loyalty, now need to adapt to fluid, episodic, and context-driven interactions. Biomedical tools, such as mobile diagnostics and predictive analytics, generate nonlinear touchpoints that challenge traditional CRM funnels [25]. Although CRM provides useful tools for long-term engagement, many studies rely heavily on commercial analogies, neglecting health care–specific factors such as emotional trust, reactions to medical errors, and the maintenance of continuity of care during crises.

#### Digital Branding and Trust as Ethical Infrastructure

Trust in digital health systems goes beyond user experience; it is built on transparency, data sovereignty, and clarity in algorithmic operations. Mohamed [27] emphasized that branding must now communicate not only usability but also ethical intent. However, most studies treat trust as an outcome rather than a process. There is a need for more in-depth, culturally responsive frameworks that examine how trust develops across various social and institutional contexts.

#### Adoption Beyond Early Adopters

Innovation adoption is often interpreted through the lens of early adopters, but this perspective overlooks the systemic factors that lead certain populations to resist digital health tools [33]. These include limited infrastructure, historical mistrust, and mismatched cultural values. The existing literature lacks a pluralistic adoption model that reflects the social, historical, and geographic nuances that influence adoption behavior.

#### The Ethical Ambiguity of Nudging

The conceptual model often presents digital tools, such as AI platforms, wearables, and CRM systems, as if they will inherently empower patients, foster engagement, and enhance autonomy. Although these technologies hold great promise, including personalized care, deeper engagement, and patient co-creation, this view risks overlooking important challenges, including bias, inequality, and subtle forms of coercion (Table 7). Patients may experience technology fatigue, struggle with data misinterpretation, or face structural inequities that limit access and benefits. At the same time, behavioral economics and nudging strategies, though effective in shaping healthier choices, blur the line between ethical persuasion and unintended coercion. In high-stakes health contexts, design elements intended to encourage positive behaviors can inadvertently pressure or manipulate patients, undermining trust. However, few studies critically distinguish between user-centered design that supports autonomy and subtle mechanisms of control that erode it. For the PAO model to develop into a strong framework, it must include a more balanced view—one that recognizes both the potential of digital tools and the ethical and structural risks they pose. This balance will be key for creating frameworks that empower patients without sacrificing ethical safeguards, equitable access, agency, or trust.

**Table 7.** Empowering potential and risks of digital tools in the patient as an organization (PAO) model, as developed by the authors based on concepts from digital health, behavioral economics, and health care marketing literature.

Digital tools or strategies	Potential benefits	Associated risks
AI^a^ platforms and predictive analytics	Deliver personalized recommendations, enable anticipatory care, and support clinical decision-making	Risk of algorithmic bias, misinterpretation of complex data, and over-reliance on automated outputs
Wearables and biosensors	Provide real-time monitoring, support self-management, and allow early detection of health concerns	Can lead to technology fatigue, data overload, and unequal access due to cost or connectivity
CRM^b^ systems in health care	Strengthen patient-provider relationships, tailor communication, and encourage proactive engagement	May reduce patients to “customers,” raise privacy concerns, or foster dependency on system-generated prompts
Digital nudging and behavioral economics	Encourage healthier behaviors, improve adherence, and reinforce positive routines	Raise ethical concerns about manipulation, risk of coercion in high-stakes decisions, and potential erosion of autonomy
Participatory platforms and co-creation	Promote shared decision-making, build trust, and empower patients as partners in care	Digital literacy gaps, exclusion of marginalized groups, and uneven levels of participation

a AI: artificial intelligence.

b CRM: customer relationship management.

#### From Information to Empowerment: Revisiting the Information-Motivation-Behavioral Skills Model

The information-motivation-behavioral skills (IMB) model is evolving from an educational tool into an empowerment framework, supported by AI-driven guidance systems, decision aids, and adaptive learning tools [30]. However, questions persist: Who determines which information is relevant? How is motivation culturally constructed?

There is a need for more critical, reflexive research that challenges normative assumptions about information delivery and authority.

#### Feedback and Co-Creation: From Input to Shared Power

Co-creation platforms and feedback mechanisms are central to participatory health care, but many current models stop at surface-level input. Without mechanisms for integrating patient feedback into system design and decision-making, participation risks becoming symbolic [31]. Few studies distinguish between procedural engagement and substantive influence, leaving a gap in conceptualizing truly collaborative health systems.

Together, these insights suggest a paradigmatic shift in how we define and interact with the digital patient. The PAO model, when fully realized, reframes the patient as a co-manager, system shaper, and strategic partner in care. This review clarifies the field’s theoretical boundaries and proposes an interdisciplinary foundation integrating biomedical engineering, ethical marketing, behavioral science, and patient co-agency. As the digital health landscape continues to evolve, this lens provides researchers, designers, and policymakers with a critical roadmap for developing ethical, inclusive, and technologically responsive systems.

#### Toward a Conceptual Contribution

The PAO model is often described as if individuals could fully take on the roles of structured organizations, overseeing strategy, governance, and operations. Although this metaphor effectively represents patient empowerment, it risks oversimplifying complex realities and may overestimate what patients can do. Unlike formal organizations, patients usually lack dedicated resources, hierarchical leadership, and institutional authority. Without a clearer definition of what “organizational behavior” means at the individual level, the PAO risks becoming more of a rhetorical device than a practical framework.

**Figure 4.**
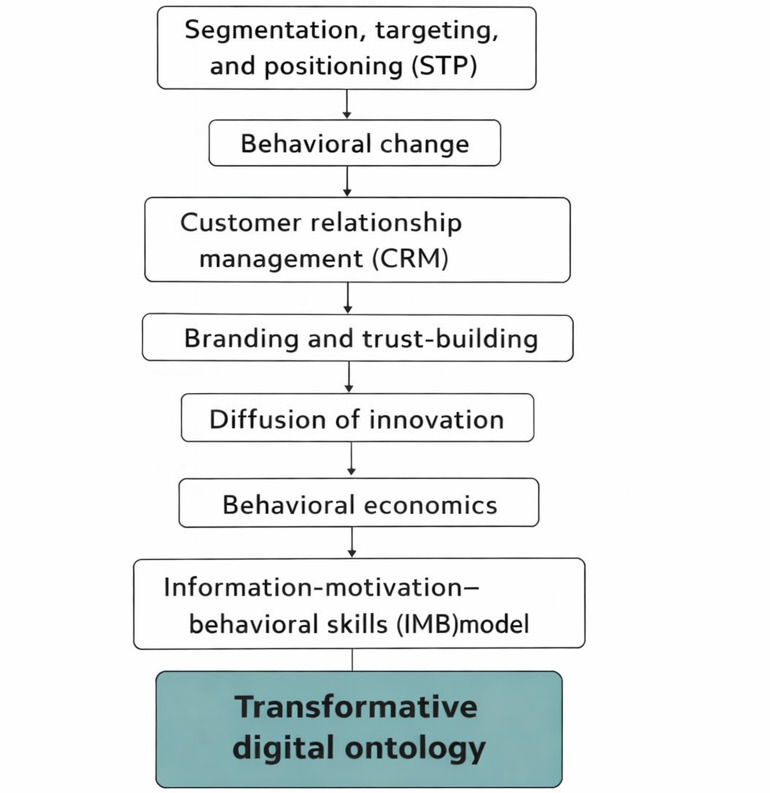
Sequential integration of marketing theories into the patient as an organization (PAO) framework, working toward a conceptual contribution of the patient as a strategic, participatory actor within a dynamic digital health ecosystem and based on the authors’ thematic synthesis of literature included in the scoping literature review.

This synthesis offers an opportunity to rethink the PAO model, not as a collection of marketing ideas but as a transformative digital framework. As shown in Figure 4, this new approach builds on a step-by-step integration of segmentation, trust-building, behavioral economics, and co-creation strategies, culminating in the development of a transformative digital ontology. Together, these mechanisms change the patient’s role from a passive recipient of care to an active, data-informed participant in health care ecosystems. Biomedical technologies are central to this shift: By combining data, autonomy, and organizational logic, they redefine what it means to be a “patient,” creating new forms of digital identity that are both empowered and connected [34].

It is important to recognize that the shift toward the PAO model is not equally accessible to all. How patients engage with digital tools is shaped by power dynamics, design decisions, access gaps, and governance structures. Acknowledging these realities does not weaken the PAO concept—it strengthens it by promoting a more inclusive and critical approach to digital health. To advance the field, research must move beyond idealized visions and explore how these dynamics play out in practice. A critical digital health perspective is essential: one that asks what technologies do, what kinds of patient roles they create, and who truly benefits. Rather than undermining the PAO model, this approach ensures it evolves ethically and equitably, with patient trust at its core. By embracing both its potential and its limitations, the PAO model can become a meaningful framework to guiding the future of patient-centered digital health care.

Table 2 highlights the potential of biomedical technologies and marketing frameworks to bring the PAO model into practice but also reveals gaps in the evidence.

#### Inference and Interpretive Insight

These findings demonstrate that the PAO model is somewhat implemented in practice, especially among digitally engaged patients. However, its application varies and is contingent on access, literacy, trust, and ethical clarity. Although digital tools are starting to promote organizational behaviors, such as self-monitoring, treated responses based on segmentation, and adaptations driven by feedback, they are primarily effective within privileged structures [35].

Participants appreciate hyper-personalization but seek equity, transparency, and human connection. These results underscore the need for interdisciplinary collaboration to ensure that digital health systems are technologically advanced, ethically sound, and socially inclusive.

#### Repositioning the Patient: Conceptual and Thematic Foundations of the PAO Model

This analysis reframed the PAO model by combining insights from biomedical innovation and marketing theory, viewing patients as active, data-driven participants in digital health systems. Traditionally, patient engagement focused on clinical outcomes or behavior change. The PAO model expands on this by positioning patients as strategic actors supported by tools such as wearables, mHealth apps, AI, and predictive analytics.

The scoping review identified 8 recurring themes where marketing concepts such as STP, CRM, behavioral economics, and co-creation intersect with biomedical technologies to create adaptive, personalized care ecosystems. Patients increasingly take on organizational roles including *strategy*, using data to set health priorities; *governance*, managing consent, trust, and accountability; and *operations*, coordinating daily care with digital tools.

Marketing analogies help clarify this shift. AI segmentation mirrors retail targeting but is used here for real-time health decisions. CRM becomes a patient-care platform, while nudging and digital branding influence health behaviors, much like tech firms shape user choices.

Although these analogies make the PAO model relatable, they rely heavily on conceptual models and pilot studies, lacking the clinical rigor expected in health care. The model’s strength lies in making new patient roles visible; its weakness lies in limited empirical support.

Ultimately, the PAO model moves beyond metaphor, presenting patients as informed, autonomous agents in digitally enabled care. However, real-world challenges, like power imbalances, access barriers, and ethical risks, remain. For the model to mature, it must blend the creativity of marketing insights with the credibility of clinical and behavioral evidence.

#### Key Research Gaps in the PAO Model and Marketing-Driven Digital Health With Biomedical Integration

##### Limited Empirical Validation of the PAO Model

Although the PAO model offers strong conceptual foundations, there is a notable lack of empirical studies demonstrating how patients engage in organizational-like behaviors using digital health technologies. The existing literature tends to focus on potential rather than observed behaviors [36]. To substantiate the PAO framework, there is an urgent need for longitudinal, ethnographic, and practice-based research that captures patient engagement across diverse cultural and clinical contexts.

##### Inconsistent Application of Marketing Theories in Health Care

Despite the established value of marketing frameworks such as STP, CRM, and behavioral marketing in other industries, their integration in health care remains fragmented. The literature review revealed a lack of standardization in the application of these theories to influence patient engagement or health outcomes. There is a research gap in developing an evidence-based framework that operationalizes these theories via biomedical technologies and ties them to measurable behavioral or clinical outcomes [37].

##### Underexplored Intersection of Health Equity and Digital Access

The review highlighted persistent health disparities rooted in socioeconomic status, geography, and digital literacy [38]. Although these structural barriers are well-documented individually, few studies explore how they intersect with the PAO framework or shape patient access to digital health tools. Future research should prioritize equity-focused design and examine how inclusive strategies can effectively close the digital divide in practice, rather than just in theory.

##### Neglected Ethical and Regulatory Dimensions of Digital Health Marketing

Ethical concerns, such as informed consent, data transparency, algorithmic bias, and digital nudging, are acknowledged across multiple sources [39]. However, in-depth theoretical engagement remains sparse. Most studies briefly address these issues without providing analytical frameworks or policy recommendations. This gap underscores the need for comprehensive ethical models that can guide the development and deployment of AI-driven marketing in health care settings.

##### Insufficient Understanding of AI’s Impact on Trust and Autonomy

Although AI offers significant potential for care personalization, the implications for patient trust, autonomy, and long-term relationships with health care providers are poorly understood [40]. The review revealed a lack of studies examining how predictive analytics influence user experience, clinical decision-making, or interpersonal dynamics in digital care environments. Responsible AI implementation requires empirical research that evaluates these relational dimensions.

##### Lack of Empirical Work on Nudging and Behavioral Economics

Behavioral economics and digital nudging are frequently cited as promising tools for influencing health behaviors [41]. However, the literature offers few empirical studies on their effectiveness in complex or high-stakes medical decisions. Experimental designs and real-world field studies are necessary to investigate how interface design, default options, and incentive framing influence behavior, particularly when ethical boundaries are being tested.

##### The Need for an Integrated, Evidence-Based Framework

Perhaps the most critical gap identified was the lack of a comprehensive PAO framework that cohesively combines marketing theory, behavioral psychology, and biomedical innovation. Current research is scattered across disciplinary silos and lacks a unified theory that can capture the complexity of digitally enabled, ethically nuanced patient engagement [42]. Filling these gaps requires a cross-disciplinary approach that is both essential and collaborative. Researchers must go beyond conceptual enthusiasm and move toward empirical validation, creating inclusive, ethically sound systems that empower patients without compromising trust or equity [43]. Only then can the PAO model develop into a practical, evidence-based foundation for personalized and participatory digital health care.

This study revealed a powerful shift underway in health care: Patients are no longer just following care plans; they’re actively shaping them. With the support of digital tools like wearables, mobile apps, and AI-driven platforms, many are setting health goals, tracking their progress, and making informed decisions. These behaviors closely resemble how organizations operate [44].

The scoping review showed that technologies are increasingly infused with marketing strategies like personalization, segmentation, CRM, and digital nudging. In interviews, these concepts came to life. Patients were not just using apps to manage symptoms; they were navigating complex decisions, often independently, guided by digital feedback.

However, this transformation isn’t universal. Although some patients found empowerment, others faced barriers, limited digital access, low tech literacy, or distrust in AI. Alongside enthusiasm, participants expressed concerns about losing human touch, data misuse, or feeling subtly manipulated by digital nudges [45].

These tensions reveal a deeper truth: The future of health care isn’t just about technology; it’s about how that technology is designed, delivered, and experienced. Patients want digital tools that support, not replace, human care. Many envisioned a *blended model*—one where empathy and innovation go hand in hand [46].

Ultimately, the PAO model is no longer just a metaphor. It’s emerging in real life but unevenly. To realize its full potential, future systems must be co-created with patients, grounded in trust, and built for equity not just efficiency.

This research shows that a quiet transformation is happening in medicine. Patients are no longer just recipients; they are now active participants in managing their health experiences. Through technologies such as wearables, apps, and AI platforms, patients are taking control and making real-time decisions tailored to their needs [47]. As a result, these patients are assuming responsibilities that are increasingly like self-management and self-organization.

However, this shift involves more than just technology. It also depends on how people feel, how they trust, whether they think they can maintain control, and whether they believe the systems support them. Although some tools, such as digital nudging and CRM reminders, help patients stay on track, others can make them feel anxious. Concerns about data privacy, algorithmic data misuse, and the loss of the human touch are common.

What is clear is that this transformation has great potential if approached thoughtfully. Empowerment must be balanced with protection. For the PAO model to guide future care, it needs to reflect what technology can do and what people want to achieve.

### Comparison With Prior Work

Prior work in digital health has practically proven escalating patient engagement with the assistance of vehicles such as wearables, mHealth apps, and AI-driven platforms. Ahadzadeh et al [24], for example, examined how the integration between the health belief model and the technology acceptance model dictated patient behavior in digital environments. In contrast, Rongkavilit et al [30] applied the IMB model to study medication behavior in teenagers infected with HIV/AIDS. These indicate the potential of digital tools to enhance motivation, information access, and behavioral skills [30]. These, however, primarily depict patients’ empowerment resulting from educational exposure or risk awareness, with little attention to patients as agency-rich actors with the potential for system-level agency.

### Reframing Empowerment Within the PAO Model

In addition, this research contributes to the expanding body of digital health literature, as it recasts this concept of empowerment through the PAO framework. This indicates that, as part of this paradigm, patients are not just receiving and using digital health technology but are strategic and savvy about their own data, as would be expected of organizations, not humans.

Both personalization and nudging have been touted as means of improving engagement, yet, as we explored through interviews, a more nuanced picture is emerging. Concerns about lack of transparency and accountability, as well as manipulation, have been voiced by patients, emphasizing that patient empowerment is more than mere behavior—it is ethical as well [48].

### Key Contributions

#### Adding Patient Organizational and Strategic Roles as an Expansion of the Idea of Empowerment

Behavior model integration (eg, HBM, IMB, or CRM) into a multilevel, ethics-focused PAO framework addresses structural inequality and moral controversies, especially for marginalized user communities, by providing practical validation through patient experiences that reveal both the potential and the vulnerabilities of technology.

#### Structural Inequities

There is inequality in access to digital care. Some participants, especially those lacking sufficient digital literacy and computer access, experienced identifiable barriers described under the “structural barriers” code (Table 5), which is encapsulated under inequities in digital empowerment (Table 6). Such barriers, as identified in Figure 3, lie at the intersections of trust, accessibility, and ability, emphasizing that care systems should be built for all and not just for digital sophisticates.

#### Trust, Ethics, and Digital Engagement

By contrast, trust was a motivator and an inhibitor of engagement. Doubts and reservations about data usage or about algorithms and nudging, coded under trust and ethical friction (Table 5), often revolve around ethical issues that have been aggregated into the theme of negotiating trust and ethical transparency (Table 6), emphasizing that, although medical systems need to be efficient, they should be transparent and eminently explainable and that patient autonomy must be respected.

#### Finding Meaning and Connection

Despite this momentum in confidence in digital technology, one key takeaway was evident—the need for emotional connection remained. Apps and AI technology might be incredibly convenient and informative, but in no way could these technologies provide emotional understanding, particularly with emotional experiences and more complex medical choices. The best possible solution continued to be that found within the process of blended care (see “blended care as the preferred future” in Figure 3), where digital technology interacted with emotional connection and interpersonal trust that was found to be absolutely critical to the process of care in the digital age.

### Strengths and Weaknesses

A key strength of this study was its mixed methods approach, which combines a broad literature review with rich qualitative insights. The use of grounded theory enabled the emergence of nuanced, real-world themes that reflect the diversity of patient experiences.

However, the study was limited by a relatively small and context-specific sample in the qualitative phase. Broader validation across diverse populations and health care systems will be needed to assess generalizability. Additionally, the scoping review was conceptual in nature and did not include a formal risk-of-bias assessment.

### Contributions to Theory and Practice

This study makes several contributions to the evolving digital health literature. It *redefines patient empowerment* as a multilevel, ethically grounded process that includes digital skills, trust-building, and system-level support. It integrates *behavioral models* (eg, health belief model, CRM) with real-time technologies to understand how patients engage with care dynamically. It adds patient perspectives on *equity, ethics, and lived experience*, which are often missing from technologically focused research. It validates the PAO model through empirical data, showing how patients actively manage care but also struggle with ethical ambiguity and structural barriers.

### Future Directions

Future studies should consider equity-oriented design and co-creation as well as research into trust, behavior, and adoption over time or other studies on ethical metrics for nudging, personalization, and AI adoption and use in patient care and outcomes today. It is imperative that, in the future, a PAO model be one of innovation, grounded in patients’ experiences, anxieties, and values.

To ensure that the PAO model becomes an empirical reality and shifts from theoretical to practical application, the following should be prioritized in subsequent research: (1) equity-informed design: design technologies to be accessible to everyone; (2) co-creation with patients: engage patients in the design process, evaluation, and governance of digital health applications; (3) transparent and ethical infrastructures: develop consent dashboards and explainable AI; and (4) longitudinal study research: study digital behavior trust processes over time.

### Conclusion

This paper examined another important change that has come about in the health care sector. Patients are no longer passive receivers of health care but are actively participating in it with the use of technology such as wearables, health apps, and AI platforms. Technology is giving people the capacity to take charge of their health care choices [49].

We conducted our study through the review of existing literature and in-depth interviews to explore how the application of marketing approaches, such as personalization, nudging, and CRM, is integrated with digital health platforms. The PAO conceptual framework provides valuable insights to understand this transformation and recognizes that patients are “consumers” no more but act like strategic actors who use data to manage health just like any other organizational entity.

What our research tells us is that, for some patients, particularly those with robust digital connectivity and savvy, this scenario has already begun to come to fruition. Many of the patients surveyed consider themselves to be planners or “health managers,” utilizing digital feed-forward and tracking applications to shape decisions. However, such technologies are certainly not ubiquitous. Members from underserved groups presented authentic challenges to digital health adoption, such as connectivity difficulties and unfamiliarity with AI applications.

Additionally, there are other ethical considerations that must be considered. Although data personalization and nudging can be beneficial to patient health outcomes, there are worries about transparency or manipulation and autonomy if patients do not fully understand algorithmic influences [50].

Despite these challenges, the PAO model provides significant insight into what is happening to the role of the patient. Rather than viewing it as something that is complete and finished—something that needs to be applied—the best way to look at it is to realize that it can be seen as more of a thought process about the future of health care that must allow for adjustment and change.

In the end, this model encourages us to reconsider the role of patients—be it within health care as recipients of care but also as active contributors to the creation thereof. With such caution and care taken in its development, the PAO approach might lead to a more participatory and trustable future in health. Only then can we ensure that digital health is not just innovative but also inclusive, trustworthy, and deeply human.

## Supplementary material

10.2196/77115 Checklist 1 PRISMA-ScR checklist.

10.2196/77115 Checklist 2 COREQ checklist.

## Abbreviations

AI: artificial intelligence
CRM: customer relationship management
IMB: information-motivation-behavioral skills
mHealth: mobile health
PAO: patient as an organization
PRISMA-ScR: Preferred Reporting Items for Systematic Reviews and Meta-Analyses Extension for Scoping Reviews
RQ: research question
STP: segmentation, targeting, and positioning
WHO: World Health Organization
