# Supplementary material for: Conceptual Model for the Integration of Marketing Strategies and Biomedical Innovation in Patient-Centered Care: Mixed Methods Study
Source: JMIR Biomed Eng. 2026 Jan 6;11:e77115. doi: 10.2196/77115 (PMC12772582; doi:10.2196/77115)
Supplement: Checklist 1 [file biomedeng-v11-e77115-s001.docx]

**Supplementary Table S1. PRISMA-ScR Checklist**

*Preferred Reporting Items for Scoping Reviews and Meta-Analyses extension for Scoping Reviews (PRISMA-ScR)*

| **Section / Topic** | **PRISMA-ScR Item** | **Where Reported in Manuscript** |
| --- | --- | --- |
| **Title** | Identify as a scoping review | Title page; Abstract |
| **Abstract** | Provide a structured summary | Abstract (pp. 2–3) |
| **Introduction** | Rationale: Why a scoping review approach is appropriate | Introduction (pp. 4–5) |
|  | Objectives: Clear statement of review objectives | Introduction (p. 5) |
| **Methods** | Protocol & registration | Not registered; noted as limitation (p. 7) |
|  | Eligibility criteria | Methods (pp. 7–8) |
|  | Information sources | Methods (p. 7) |
|  | Search strategy | Methods; Supplementary Appendix |
|  | Selection of sources | Methods (p. 8); Figure 1 (PRISMA-ScR flow diagram) |
|  | Data charting process | Methods (p. 9) |
|  | Data items | Methods (p. 9) |
|  | Critical appraisal of sources (optional in ScR) | Not conducted; rationale provided (p. 11) |
|  | Synthesis of results | Methods (pp. 10–11) |
| **Results** | Selection of sources | Results (p. 12); Figure 1 |
|  | Characteristics of sources | Results; Table 1 |
|  | Results of individual sources | Results (pp. 12–15) |
|  | Synthesis of results | Results (pp. 12–16) |
| **Discussion** | Summary of evidence | Discussion (pp. 16–18) |
|  | Limitations | Discussion (p. 19) |
|  | Conclusions | Conclusion (p. 20) |
| **Funding** | Source of funding | Acknowledgments (p. 22) |

**Inclusion and Exclusion Criteria (Scoping Review)**

To ensure transparency, consistency, and methodological clarity, inclusion and exclusion criteria were clearly defined and applied throughout the article selection process in accordance with the PRISMA-ScR framework. These criteria guided both the abstract and full-text screening stages and are illustrated in Figure 1 (PRISMA-ScR flow diagram).

Inclusion Criteria
Studies were eligible if they met the following conditions:

- Published in peer-reviewed journals or academic monographs between 2014 and 2025, with particular emphasis on the most recent five years.
- Addressed intersections among biomedical technology, healthcare marketing, digital transformation, and organizational behavior.
- Contributed a clear theoretical model, empirical framework, or system-level insight related to themes such as patient activation, AI-driven personalization, or behavioral change strategies in healthcare.

**Exclusion Criteria**Studies were excluded if they:

- Focused solely on biomedical or clinical outcomes without reference to organizational, behavioral, or marketing perspectives.
- Were non-English publications, to avoid issues of translation and potential loss of conceptual precision.
- Consisted of non-peer-reviewed materials, such as white papers, editorials, opinion articles, or conference abstracts lacking methodological transparency.

By applying these structured criteria, the review ensured that the included sources reflected the interdisciplinary scope of the scoping review while upholding academic standards of reliability, conceptual relevance, and thematic alignment.
