# Supplementary material for: Conceptual Model for the Integration of Marketing Strategies and Biomedical Innovation in Patient-Centered Care: Mixed Methods Study
Source: JMIR Biomed Eng. 2026 Jan 6;11:e77115. doi: 10.2196/77115 (PMC12772582; doi:10.2196/77115)
Supplement: Checklist 2 [file biomedeng-v11-e77115-s002.docx]

**Supplementary Table S2. COREQ Checklist**

*Consolidated Criteria for Reporting Qualitative Research (COREQ): 32-item checklist for interviews and focus groups*

| **Domain** | **Item** | **Where Reported in Manuscript** |
| --- | --- | --- |
| **Domain 1: Research team & reflexivity** | Interviewer/facilitator | Methods (p. 10) |
|  | Credentials | Methods (p. 10) |
|  | Occupation of interviewer | Methods (p. 10) |
|  | Gender of interviewer | Methods (p. 10) |
|  | Experience/training | Methods (p. 10) |
| **Domain 2: Study design** | Methodological orientation | Grounded theory; Methods (p. 10) |
|  | Sampling | Purposive; Methods (p. 9) |
|  | Approach to participants | Recruitment by email/contacts; Methods (p. 9) |
|  | Sample size | 18 participants; Methods (p. 9) |
|  | Non-participation | Number approached vs. interviewed; Methods (p. 9) |
|  | Setting of data collection | Online/healthcare context; Methods (p. 10) |
|  | Presence of non-participants | Not applicable |
|  | Description of sample | Demographics/roles; Results; Table 2 |
|  | Interview guide | Semi-structured; Methods (p. 10) |
|  | Repeat interviews | Not conducted |
|  | Audio/visual recording | Audio-recorded with consent; Methods (p. 10) |
|  | Field notes | Taken; Methods (p. 10) |
|  | Duration | 45–60 min; Methods (p. 10) |
|  | Data saturation | Reported; Methods (p. 10) |
|  | Transcript return | Not returned; noted as limitation |
| **Domain 3: Analysis & findings** | Number of coders | Two; Methods (p. 11) |
|  | Coding framework | Open, axial, selective coding; Methods (p. 11) |
|  | Theme derivation | Grounded theory; Methods (p. 11) |
|  | Software | NVivo/manual; Methods (p. 11) |
|  | Participant checking | Not conducted; limitation |
|  | Quotations | Participant quotes included; Results (pp. 13–15) |
|  | Data consistency | Yes; triangulation; Methods (p. 11) |
|  | Clarity of major themes | Results (pp. 12–16) |
|  | Clarity of minor themes | Results (p. 15) |
|  | Return of findings to participants | Not applicable |
